# Sarcopenia and Heart Failure

**DOI:** 10.3390/nu12010211

**Published:** 2020-01-14

**Authors:** Francesco Curcio, Gianluca Testa, Ilaria Liguori, Martina Papillo, Veronica Flocco, Veronica Panicara, Gianluigi Galizia, David Della-Morte, Gaetano Gargiulo, Francesco Cacciatore, Domenico Bonaduce, Francesco Landi, Pasquale Abete

**Affiliations:** 1 Department of Translational Medical Sciences, University of Naples Federico II Naples, 80131 Naples, Italy; checcocurcio@libero.it (F.C.); gianluca.testa@unimol.it (G.T.); liguorilaria@gmail.com (I.L.); papillomartina@gmail.com (M.P.); veronica.fabry@gmail.com (V.F.); v.panicara@gmail.com (V.P.); gianluigi.galizia@icsmaugeri.it (G.G.); francesco.cacciatore@unina.it (F.C.); bonaduce@unina.it (D.B.); 2 Department of Medicine and Health Sciences, University of Molise, 86100 Campobasso, Italy; 3 IRCCS Salvatore Maugeri Foundation, Scientific Institute of Veruno, 28010 Novara, Italy; 4 Department of Systems Medicine, University of Rome Tor Vergata, 00100 Rome, Italy; david.dellamorte@uniroma2.it; 5 San Raffaele Roma Open University, School of Medicine, 00100 Rome, Italy; 6 Division of Internal Medicine, AOU San Giovanni di Dio e Ruggi di Aragona, 84121 Salerno, Italy; gaetanogargiulo@libero.it; 7 Department of Geriatrics, Neurosciences and Orthopedics, Catholic University of the Sacred Heart, 00100 Rome, Italy; francesco.landi@unicatt.it

**Keywords:** sarcopenia, heart failure, elderly, cachexia, malnutrition, physical activity

## Abstract

Modifications of lean mass are a frequent critical determinant in the pathophysiology and progression of heart failure (HF). Sarcopenia may be considered one of the most important causes of low physical performance and reduced cardiorespiratory fitness in older patients with HF. Sarcopenia is frequently misdiagnosed as cachexia. However, muscle wasting in HF has different pathogenetic features in sarcopenic and cachectic conditions. HF may induce sarcopenia through common pathogenetic pathways such as hormonal changes, malnutrition, and physical inactivity; mechanisms that influence each other. In the opposite way, sarcopenia may favor HF development by different mechanisms, including pathological ergoreflex. Paradoxically, sarcopenia is not associated with a sarcopenic cardiac muscle, but the cardiac muscle shows a hypertrophy which seems to be “not-functional.” First-line agents for the treatment of HF, physical activity and nutritional interventions, may offer a therapeutic advantage in sarcopenic patients irrespective of HF. Thus, sarcopenia is highly prevalent in patients with HF, contributing to its poor prognosis, and both conditions could benefit from common treatment strategies based on pharmacological, physical activity, and nutritional approaches.

## 1. Introduction

Heart failure (HF) is defined by the European Society of Cardiology (ESC) guidelines as “a clinical syndrome characterized by typical symptoms (e.g., breathlessness, ankle swelling and fatigue) that may be accompanied by signs (e.g., elevated jugular venous pressure, pulmonary crackles and peripheral edema) caused by a structural and/or functional cardiac abnormality, resulting in a reduced cardiac output and/or elevated intra-cardiac pressures at rest or during stress” [1]. HF affects up to 2% of the population in developed countries. It leads to morbidity, institutionalization, and death, and it poses a major challenge to healthcare institutions worldwide [1]. Noticeably, the prevalence of Heart Failure (HF) increases dramatically with age. Much of this rise primarily stems from the implementation of more effective therapeutics that have augmented life expectancy in individuals with HF [2]. Therefore, a large number of patients older than 65 experience clinical problems, including frailty status, which was never traditionally considered as a relevant issue but has become a major clinical challenge for physicians nowadays.

In particular, the role of sarcopenia, i.e., a reduction of strength and mass muscle, has recently received intense analysis in patients with HF so that changes in muscle function and composition are considered critical determinants in the pathophysiology and progression of this condition. In fact, in addition to cardiac dysfunction and reduced cardiac output, which are obviously required for defining HF, age-related muscle decline overlapping with “cardiac skeletal myopathy” may be considered the most important cause of low physical performance and reduced cardiorespiratory fitness in older patients with HF [3].

Thus, sarcopenia may contribute to the poor prognosis of HF patients, although the pathophysiology of the relationship between these two conditions is unclear. In fact, sarcopenia and HF appear to present several similar pathogenetic pathways, and therefore, they could benefit from a common therapeutic approach.

## 2. Muscle Changes in HF: Cachexia or Sarcopenia?

Cachexia may be defined as a multifactorial syndrome characterized by severe body weight, fat, and muscle loss and increased protein catabolism due to underlying disease(s) [4]. The term sarcopenia, on the other hand, indicates an age-related loss of skeletal muscle quantity or quality and a decline in muscle strength and/or physical performance not necessarily associated with weight loss [5].

von Haehling describes the “wasting continuum in HF.” He suggests that since sarcopenia predominantly affects postural rather than non-postural muscles, and cachexia leads to a loss of fat tissue and weight loss, the “wasting continuum in HF” implies that skeletal muscle is lost earlier than fat tissue and, therefore, sarcopenia precedes cachexia [6]. The causes of cachexia are multifactorial and may cause neurohormonal derangements, poor nutrition and malabsorption, impaired calorie and protein balance, anabolic hormone resistance, prolonged immobilization, and physical deconditioning, which is the typical condition of an older HF patient [1].

Although widely overlapping and difficult to recognize, these two conditions are highly prevalent in HF. In an analysis of the SICA-HF (Studies Investigating Comorbidities Aggravating Heart Failure), evaluating 200 individuals with HF with reduced ejection fraction (HFrEF, 68.8%) or HF with preserved ejection fraction (HFpEF, 31.2%), muscle wasting was present in about 32% of the patients, of whom 30 presented with sarcopenia alone (14.4%), 25 with cachexia alone (12.1%), and 14 with sarcopenia and cachexia together (6.7%) [7]. Similar from the clinical point of view, cachexia and sarcopenia have very different histopathological features as well as different physiopathological characteristics. Age-related changes in skeletal muscle that occur in the sarcopenic myopathy are quite different from those found in “cardiac skeletal myopathy.” Sarcopenia is characterized by atrophy of the muscle fibers, which concerns type II compared to type I muscle fibers, and by a progressive denervation and re-innervation process, likely secondary to a chronic neuropathic process resulting in a loss of motor unit. The infiltration of fat and connective tissue is another important element of reduced muscle quality, as well as decrease in blood flow, evaluated by capillary density [8,9]. Patients with severe HF have dissimilar histological abnormalities: In these subjects it has been found that before atrophy, a fiber type inversion occurs with a lower percentage of slow-twitch type I fibers and a higher percentage of fast-twitch type II. Besides the deposition of fibrotic and adipose tissue, intrafibrillar edema related to the secretion of NT-proBNP has also been described. The reduction of capillary density is another common finding closely related to fibrosis and microcirculation alterations in response to reduced cardiac output [10]. In early stages of cardiac cachexia, a progressive rise in the number of intramyocellular lipid droplets is common, reflecting an imbalance between fatty acid supply and utilization, also linked to alterations in the number and mitochondrial function. As the cachectic process progresses, a generalized atrophy of the muscle fibers is observed which, unlike sarcopenia, does not spare type I fibers and is associated with a loss of adipose and bone tissue [11].

Importantly, muscle wasting conditions in HF also have different pathogenetic imprints. In sarcopenia, energy expenditure is typically not increased or even reduced, while in cachexia energy cost, resulting from an accelerated hypermetabolic state, is increased. Systemic inflammation is also increased to a much greater degree in cachexia when compared to sarcopenia [12,13].

**Figure 1:**
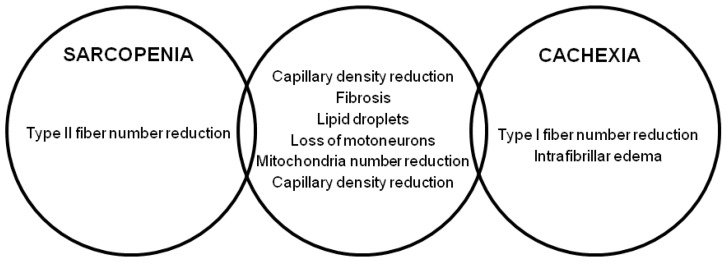
Skeletal muscle histological alterations in sarcopenia and cachexia adapted from von Haehling et al. [4].

Figure 1 shows the differences between sarcopenic and cachectic muscle.

## 3. From Heart Failure to Sarcopenic Muscle

**Figure 2:**
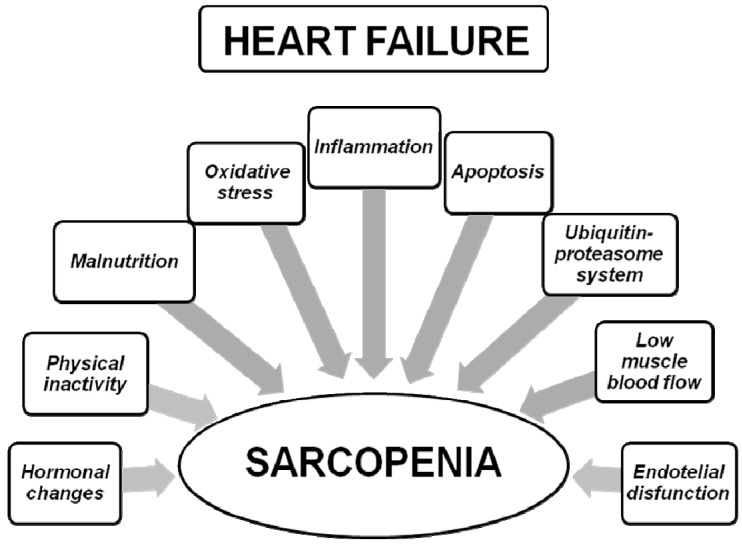
Factors related to heart failure, potentially leading to sarcopenia.

As shown in Figure 2, skeletal muscle dysfunction in sarcopenia may be determined by several factors related to HF. As is well known, HF is characterized by the presence of several modifications including hormonal changes, malnutrition and malabsorption, inflammation and oxidative stress, apoptosis, overactivation of ubiquitin-proteasome system, physical inactivity, low muscle blood flow, and endothelial dysfunction. All of these HF-related factors may contribute to the development of sarcopenia in HF patients.

### 3.1. Hormonal Changes

Clinicians initially viewed heart failure in a “hemodynamic” model as a result of abnormalities of the pumping capacity of the heart and excessive peripheral vasoconstriction [14]. Later on, this approach was substituted by the “neurohormonal model,” in which heart failure progresses as a result of the overexpression of biologically active molecules capable of exerting deleterious effects on the heart and circulation in response to a reduction of cardiac output [14]. It is interesting to note that the activation of these neuro-hormonal responses has been associated with the development of sarcopenia and muscle wasting.

In animal models, Angiotensin II infusion can induce muscle wasting via altered insulin-like growth factor-1 (IGF-1) signaling, increased apoptosis, enhanced muscle protein degradation breakdown by overactivation of the ubiquitin proteasome system (UPS), and reduced appetite [15,16]. Other preclinical studies showed that the administration of ACE-inhibitors or angiotensin II type 1 receptor blockers (ARBs) counteracts these effects, reducing the extent of myocyte apoptosis and improving nitric oxide (NO) signaling and the expression of the mammalian target of rapamycin (mTOR) in old rats [17]. Chronic adrenergic stimulation, characteristic of HF, is an important contributor to muscle dysfunction due to aberrant mitochondrial function. In this respect, patients treated with beta-blockers appear to confer some degree of skeletal muscle mitochondrial protection [18]. Sustained sympathetic activity is linked with excessive oxidative stress further contributing to mitochondrial damage, muscular protein dysfunction, and degradation. Angiotensin-II, epinephrine, and norepinephrine can, moreover, cause endothelial dysfunction, heterogeneity of blood flow, and lower capillary density, all linked with sarcopenia genesis [19].

A decline in anabolic hormones has been described in HF [20]. In parallel, age-related decreases in growth hormone [GH] and insulin-like growth factor-1 (IGF-1) levels are related to reduced muscle mass and function leading to poor physical performance and sarcopenia [21]. However, attempts to manage sarcopenia through IGF-1 and GH supplementation have produced conflicting results [22]. Low testosterone levels are a common finding in HF patients and contribute to the progression of cardiac dysfunction through altered peripheral vascular resistance, increased cardiac afterload, and decreased cardiac output [23]. A decrease in testosterone levels is also associated with adverse outcomes, including muscle mass loss and functional impairment [24]. Elderly patients with HF usually have lower ghrelin levels, which is a peptide produced primarily in the fundus region of the stomach with pleiotropic actions which includes regulating appetite, promoting food intake, and growth hormone release [25]. Thus, the decreased level of ghrelin is associated with the occurrence of sarcopenia.

Myostatin (MYO) is a member of the transforming growth factor β family and exerts a powerful action as a negative muscle growth regulator, confirmed by the evidence that MYO-knockout mice show abnormal muscle hypertrophy, while MYO overexpression leads to severe atrophy [26]. In consideration of its biological significance, intense research on the role of MYO in the development of sarcopenia has been carried out, and it has been demonstrated that MYO gene expression is increased in older men relative to younger individuals and MYO inhibition enhances the effects of exercise on performance and metabolic outcomes in animal models [27]. Interestingly, MYO has also been shown to be significantly upregulated in HF patients [28], where it exerts a myocardium anti-hypertrophic, but pro-fibrotic effect [29]. Heineke et al. [30] demonstrated that MYO released from cardiomyocytes induced muscle atrophy in HF.

### 3.2. Malnutrition and Malabsorption

Patients with severe HF frequently suffer from a gastroenteropathy secondary to intestinal edema which causes malabsorption and anorexia. Anorexia, independently associated with decreased muscle mass and strength [31,32], is a common symptom in HF because it is also related to dysgeusia and nausea, frequent side effects of drugs commonly prescribed in this disease (i.e., digoxin, angiotensin-converting enzyme (ACE) inhibitors, β-blockers, and diuretics). This list can also be responsible of a loss of nutrients through urination [33]. In summary, in HF, patients’ insufficient intake or absorption of primary nutritional elements or their loss can contribute to a negative balance between energy demand and expenditure, leading to a catabolic state and causing protein-energy malnutrition.

### 3.3. Inflammation and Oxidative Stress

An increase in inflammatory markers was found in patients with HF, a condition characterized by chronic low-level inflammation, which may exert sustained effects not only on cardiovascular function but also on skeletal muscle, representing a fundamental crossing point between HF and sarcopenia. Inflammation indeed promotes muscle atrophy and elevated levels of inflammatory markers. Tumor necrosis factor-alpha, C-reactive protein, and interleukin-6 have been correlated with a decline in muscle mass and strength, suggesting a direct involvement in sarcopenia pathogenesis [34,35]. These molecules have shown capacity to induce apoptosis and proteolysis and inhibit the transcription of gene coding for the structural protein of muscle. Inflammatory cytokines are furthermore linked with activation of the UPS and may induce anorexia and lipolysis, thus contributing to sarcopenia and even more to cachexia [36].

Reactive oxygen and species (ROS) are produced by several endogenous and exogenous processes, and their negative effects are neutralized by antioxidant defenses. Oxidative stress occurs from the imbalance between ROS production and these antioxidant defenses. The oxidative stress theory of aging is based on the hypothesis that age-associated functional losses are due to the accumulation of ROS-induced damages. At the same time, oxidative stress is involved in several age-related conditions including sarcopenia and HF [37,38]. Elevated levels of oxidative stress markers have been indeed shown in HF patients and have been correlated with reduced exercise tolerance and indices of worse prognosis [39]. The underlying mechanisms could be related to low cardiac output, endothelial dysfunction, and reductions in oxygen delivery to the skeletal muscle, resulting in limited oxygen availability and subsequent reliance on anaerobic metabolism [40]. In parallel, it has been reported that sarcopenia may be triggered by ROS which can contribute to mitochondrial dysfunction and accelerate skeletal muscle damage and degeneration, particularly by interfering with the physiological excitation-contraction coupling [41,42].

### 3.4. Apoptosis

Another mechanism postulated to be linked to sarcopenia is the accelerated elimination of myonuclei through an apoptosis-like process known as “myonuclear apoptosis.” In skeletal muscle, apoptosis exhibits unique characteristics in view of the multinuclear nature of myocytes and this pathway results in fiber atrophy rather than whole cell death. Several apoptotic pathways have been linked with age-related muscle atrophy [43] and higher frequency of myonuclear apoptosis has also been found in the muscle of patients with HF relative to age-matched healthy controls [44].

### 3.5. Ubiquitin-Proteasome System (UPS)

In HF, one of the main determinants of skeletal muscle atrophy is an imbalance of myofibrillar protein levels, probably due to increased protein catabolism, primarily attributed to overactivation of the UPS pathway [45]. UPS is the primary mechanism of protein degradation and includes a multi-subunit protease that specifically degrades ubiquitin-conjugated proteins in proteasomes [46]. UPS is induced in response to myostatin/transforming growth factor-β signaling and is critically involved in the pathogenesis of sarcopenia [47]. Interestingly, UPS is also overexpressed in the skeletal muscle of patients with HF, and this rise seem to be induced by pro-inflammatory cytokines such as TNF-α, IL-6, and IL1β [48].

### 3.6. Physical Inactivity

Age-related decline in exercise capacity is an important determinant of the loss of muscle mass and strength and is recognized as one of the most effective interventions for sarcopenia [49,50]. Cardiac dysfunction in HF strongly affects cardio-respiratory fitness leading to physical inactivity and long bedrest that contributes to muscle wasting and dysfunction [51]. Furthermore, physical inactivity has been linked to most of the pathways underlying HF-associated muscle catabolism, including the UPS pathway, inflammation, and myostatin signaling [52,53].

### 3.7. Low-Muscle Blood Flow and Endotelial Dysfunction

In HF, reduction in cardiac output results in a decline in skeletal muscle blood flow, worsening muscle mass and strength. Low-muscle blood flow, as evidenced by a reduction in capillary density, is a potentially significant factor for lower muscle performance and is related to metabolic alteration with a greater reliance on glycolytic, as opposed to oxidative, pathways to produce energy [54]. HF patients indeed show substantial reduction in the ratio of oxidative type I to fast, glycolytic type II fibers. Reductions in oxygen delivery to the skeletal muscle may modulate this change in fiber type due to the resulting limited oxygen availability and subsequent reliance on glycolytic pathways [55]. In HF patients, oxygen delivery to the skeletal muscle is limited by observed reductions in microvascular vasodilatory capacity other than the reduction in capillary density. Low baseline and peak reactive hyperemia blood flow is commonly experienced in HF, suggesting that HF patients are more likely to suffer from endothelial dysfunction, which is one of the mechanisms of sarcopenia [56]. Furthermore, inadequate perfusion pressure gradient secondary to increased central venous pressures can aggravate tissue ischemia and promote lactate accumulation and muscle dysfunction.

## 4. From Sarcopenic Muscle to Heart Failure

Functional capacity is a primary concern for patients with HF. Physical performance, as assessed by peak oxygen consumption (peak VO_2_) or distance of a 6-min walk, are the most important factors in the prognosis of HF [57]. Interestingly, a weak relation between hemodynamic parameters (e.g., left ventricular filling pressure, left ventricular ejection fraction, diastolic function, and rest cardiac output) and functional capacity [58] has been described. In contrast, sarcopenia, i.e., muscle strength and mass reduction, significantly affects physical performance, evaluated as peak VO_2_. In fact, sarcopenia may lead to characteristics of physical frailty [59,60].

For this reason, Coats et al. proposed in HF patients the “muscle hypothesis” in which skeletal muscle abnormalities, including skeletal muscle atrophy, decreased oxidative fibers and enzymes, and capillary and mitochondrial volume density, can account for much of the pathophysiology and symptoms of the condition, contributing to the reduced peak VO_2_ [61].

### 4.1. Sarcopenia, Ergoreflex, and HF

A muscle metaboreceptor (ergoreceptor) contributes to the hemodynamic and autonomic responses to exercise by controlling the sympathetic, hypertensive, and hyperpnoic responses to exercise and may have a role in the vicious cycle of sympathetic activation, which is considered one of the central elements of HF pathogenesis [62]. The ergoreceptors are sensitive to work performed by exercising skeletal muscle and their signal is transmitted in the lateral spinothalamic tracts which is responsible in part for mediating adrenergic activation during exercise. The ergoreceptors cause a withdrawal of parasympathetic tone, as well as an enhancement of adrenergic activity [62]. In contrast, exercise rehabilitation may reduce sympathetic activity and improve HF outcomes by its effect on muscle function. It has been demonstrated that low skeletal muscle mass is associated with low aerobic capacity and increased mortality risk in HF patients (CARE—CR study) [63].

### 4.2. Sarcopenia, Muscle Fatigue, and Dyspnea in HF

Muscle fatigue and dyspnea are the main symptoms in HF commonly reported by patients limiting their normal daily activities. In only a few patients, dyspnoea is due to pulmonary congestion and elevation in pulmonary capillary wedge pressures, as well as fatigue linked to muscle underperfusion [64]. In this scenario it is important to consider how sarcopenia-related abnormalities in peripheral blood flow and skeletal muscle can play a key role in producing both objective limitations to exercise and in explaining the generation of the exercise-limiting symptoms of HF [65].

Interestingly, in the analysis of SICA-HF by Emami et al. while both peak VO_2_ and quadriceps strength were significantly reduced in sarcopenia and cachexia, the 6-min walking test (6MWT), hand grip strength, and quality of life were only reduced in patients with sarcopenia, suggesting that in HF sarcopenia may be responsible for a more severe impairment of functional capacity and muscle strength compared to cachexia [7].

## 5. Does a Sarcopenic Heart Exist?

The usual mechanism of cardiac adaptation to the conditions of an increase of systemic demand is cardiac hypertrophy. Physiological hypertrophy of the heart occurs in pregnancy, as well as in athletes, while pathological hypertrophy is induced by factors such as prolonged and abnormal hemodynamic stress, i.e., hypertensive state, which can lead to cardiac dysfunction [66]. Therefore, a real process of “cardiac sarcopenia,” i.e., cardiac mass modification and dysfunction, as an analogy to what happens in skeletal muscle is currently minimally investigated.

In the SICA-HF sub-study, 117 symptomatic outpatients with HFpEF were divided into three groups according to the E/e^1^ value, an indirect parameter of left ventricular diastolic pressure: ≤8, 9–14, and ≥15. Sarcopenic patients showed the highest value of E/e^1^ [>15], indicating a strict relationship between HFpEF and sarcopenia [67]. Substantial attention has focused on defining the “central” versus “peripheral” mechanisms underlying the reduced functional capacity and exercise tolerance among patients with HFpEF. In fact, while drug trials focusing on influencing cardiovascular function have not improved exercise capacity, physical training has shown to improve exercise tolerance via peripheral adaptive mechanisms, even in the absence of favorable central hemodynamic function. This suggests that peripheral limitations linked to aging-related abnormalities in body composition, i.e., sarcopenia, may play a significant role in limiting exercise tolerance, a hallmark feature of HFpEF, even more pronounced than in cardiac diastolic dysfunction [68,69,70].

**Figure 3:**
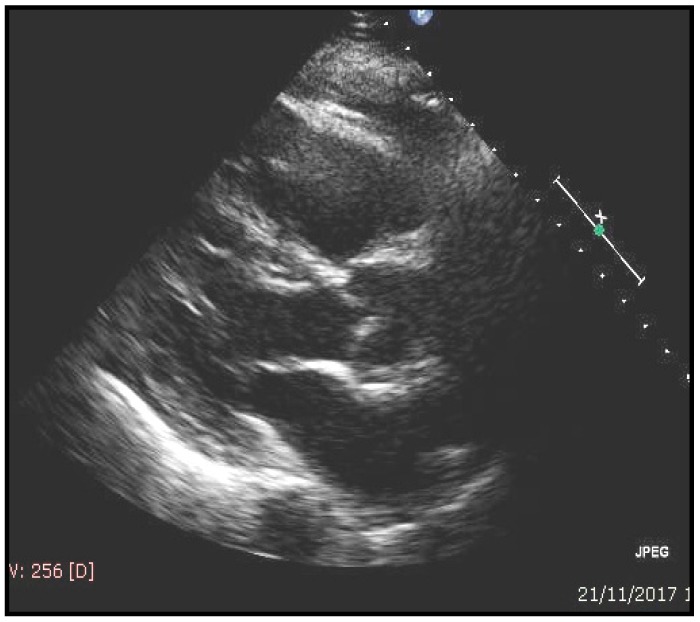
A typical example of sarcopenia and echocardiographic evaluation in an 82-year-old male patient. A reduction of strength and mass muscle is associated to “non-functional” cardiac hypertrophy (LV = left ventricular; *E/e*^1^ = echocardiographic transmitral early peak velocity (*E*) by pulsed wave Doppler over *e*^1^ (*E/e*^1^) represent a noninvasive surrogate for LV diastolic pressures for grading a diastolic dysfunction).

**Figure 4:**
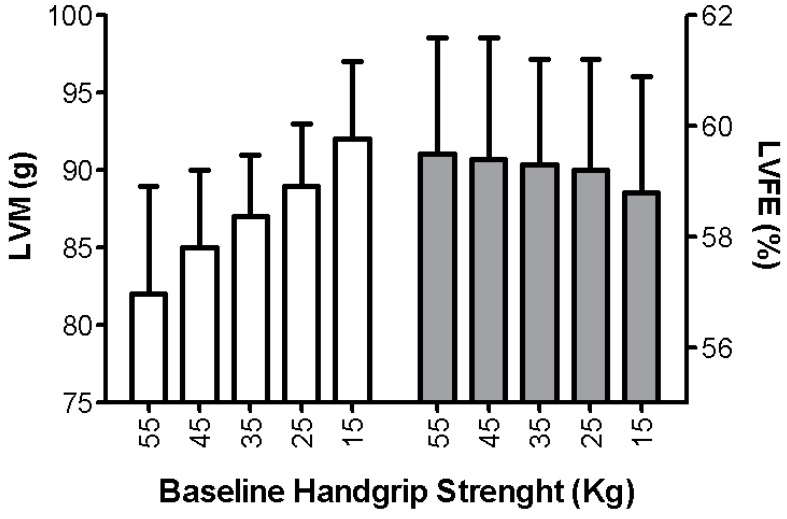
Increase of left ventricular mass [LVM, g] and reduction of left ventricular ejection fraction (LVEF, %) associated with a reduction of handgrip strength [modified by Beyer et al. [72]).

Interestingly, in the GLISTEN study on elderly hospitalized patients, sarcopenia was associated with the presence of HF in 19.4% (men = 20.2% and woman = 18.0%); in the absence of sarcopenia, HF prevalence was 16.3% (men = 13.5 and woman = 18.6%) [71]. In some cases, by performing an echocardiography study a correlation between sarcopenia and cardiac hypertrophy has been found (left ventricular mass 119 ± 14 g/m^2^ in sarcopenic vs. 105 ± 14 gr/m^2^ in not sarcopenic patients *p* = 0.02). A typical sarcopenic patient with echocardiographic evaluation is shown in Figure 3. Accordingly, previous studies have indicated an inverse correlation between handgrip strength and cardiac mass for patients at risk of sarcopenia [72]. More importantly, a reduction of muscle strength was associated with an increase in ventricular mass, together with a reduction of ejection fraction, leading to a “not-functional cardiac hypertrophy” (Figure 4).

Accordingly, the earliest description of HFpEF was primarily conceptualized as a diastolic filling impairment and only later inflammation and multimorbidity, that play a key role in the development of sarcoepnia, have been considered as elemental contributors to HFpEF development [73].

In this context, a sarcopenic heart characterized by a “not-functional hypertrophy” may be considered as an intriguing hypothesis.

## 6. Therapeutic Approach in Sarcopenic Patients with Chronic Heart Failure

Sharing several pathogenetic pathways, as illustrated above, sarcopenia and HF could therefore benefit from common treatment strategies that can simultaneously target cardiac and muscle dysfunction. First-line agents for the treatment of HF involve ACE inhibitors or beta-blockers which offer a beneficial effect on survival, and physical activity and nutritional interventions may offer therapeutic advantage in sarcopenic patients irrespective of HF.

### 6.1. Pharmacological Approach

ACE inhibitors and ARBs have been shown to possess a plethora of extracardiac effects [74], some of which may be harnessed for the management of body wasting. They possess muscle-protective properties spanning mitochondrial function, oxidative stress, insulin sensitivity, NO signaling, and local inflammation [17,75,76]. However, a recent systematic review and meta-analysis concluded that treatment with ACE-inhibitors did not significantly improve walk distance or muscle strength and function in the elderly [77]. Furthermore, it is not clear if the effects of ACE-inhibitors on physical performance are linked to direct actions on skeletal muscle or are secondary to improvements in hemodynamics. Given these contrasting findings, specifically designed trials are needed to definitively establish if ACE-inhibitors and ARBs may offer therapeutic gain in the treatment of sarcopenia and HF-related muscle wasting.

Beta-blockers, another fundamental pillar in the treatment of HF, have been reported to reduce the risk of weight loss in patients with HF. However, improvements in body weight in these patients appeared to be primarily attributable to the inhibition of lipolysis and gains in fat mass, whereas no muscle-specific effects could be demonstrated [78,79].

Aldosterone antagonists such as spironolactone, finally, may delay the progression of sarcopenia by reducing skeletal myocyte apoptosis, improving vascular endothelial function and enhancing muscle contractility [80].

### 6.2. Physical Activity Approach

Exercise is the only meaningful therapeutic approach to muscle wasting in HF that has sufficient clinical evidence. It reduces oxidative stress and the activity of the UPS [48,81], reduces pro-inflammatory cytokine expression [82], and elevates tissue expression of myostatin in skeletal muscle [83]. In addition, physical exercise may enhance vagal tone and decrease sympathetic activity, thereby improving endothelial function in CHF patients.

### 6.3. Nutritional Approach

Adequate nutritional intake or special nutritional supplementation may represent the best strategy for prevention or delay of sarcopenia, which worsens the muscle function and physical performance in patients with HF. There is evidence to support that excess protein (1.0–1.2 g/kg body weight/day) may also improve muscle mass or physical function [84]. Notably, beta-hydroxy-beta-methylbutyrate (HMB), a metabolite of the essential amino acid leucine, has been showed to affect muscle protein turnover by stimulating protein synthesis associated with its anti-catabolic action in skeletal muscle, and its supplementation can attenuate the development of sarcopenia in elderly subjects [85]. Interestingly, in addition to standard care, the administration of a high-protein oral nutritional supplement containing HMB in malnourished, older adults hospitalized for HF induced a reduction on readmission and mortality [86].

Finally, vitamin D deficiency is common in HF patients and has been associated with loss of muscle mass and impaired physical performance in elderly subjects with and without HF [87]. It is noteworthy that vitamin D influences the pathophysiology of HF by modulating the renin-angiotensin system, calcium handling, inflammation, blood pressure, and endothelial function [88]. Additionally, vitamin D supplementation may decrease serum levels of the parathyroid hormone and inflammatory cytokines (i.e., TNF-α and CRP) in HF patients, therefore its supplementation is regarded as an appealing strategy to manage sarcopenia in the setting of HF.

### 6.4. Single Therapeutic Approach for Targeting HF and Sarcopenia

Regular and tolerable levels of physical activity and/or exercise training and adequate nutritional intake or special nutritional supplementation may be the most excellent approach for prevention or delay of sarcopenia and the worsening physical performance in patients with HF [89].

## 7. Conclusions

Sarcopenia is highly prevalent in patients with HF, contributing to the poor prognosis of the disease. Even though the pathophysiology of muscle wasting in HF is complex, sarcopenia and HF appear to share several pathogenetic pathways and could benefit from common treatment strategies from a pharmacological, physical activity, and nutritional approach.

## Figures and Tables

**Table:** 

**SARCOPENIA Evaluation**	**Value**	**Normal Range**
Handgrip strength, kg	20.6	<27 kg
Muscle mass, kg/m^2^	6.2	<7
Short Performance Physical Battery	5	<5
**ECHOCARDIOGRAFIC evaluation**	**Value**	**normal range**
Septal thickness, mm	13.8	0.6–0.9
Posterior wall thickness, mm	12.9	0.6–0.9
LV end-diastolic diameter, mm	45.5	39–53
LV mass/Body surface area, g/m^2^	120.4	44–88
E/e^1^	13	<10
Ejection fraction (%)	49.5	≥55
Atrial volume/Body surface area, mL/m^2^	46.4	16–28
